# Pre-TAI protocol strategies to increase reproductive efficiency in beef and dairy cows

**DOI:** 10.21451/1984-3143-AR2019-0041

**Published:** 2019-10-23

**Authors:** José Nélio de Sousa Sales, Luiz Manoel Souza Simões, Raphael Evangelista Orlandi, Eduardo Alves Lima, Ana Paula Castro Santos, Miguel Pizzolante Bottino, Luiz Augusto Capellari Leite da Silva, José Camisão de Souza, Marcelo Maronna Dias, João Paulo Martinelli Massoneto, Luiz Antônio Scandiuzzi, Bruno Gonzalez Freitas, Bruna Martins Guerreiro, Michele Ricieri Bastos

**Affiliations:** 1 Department of Veterinary Medicine, UFLA, Lavras, MG, Brazil.; 2 Department of Animal Science, UFLA, Lavras, MG, Brazil.; 3 Genex, CRI Genética Brasil, São Paulo, SP, Brazil.; 4 Agua Preta Farm, Cocalinho, MT, Brazil.; 5 Ouro Fino Saúde Animal, Ribeirão Preto, SP, Brazil.

**Keywords:** Conception rate, timed artificial insemination, LH, P4, pre-synchronization

## Abstract

Ovulation synchronization protocols are well established in beef and dairy cows. However, the protocol response rate is around 70-90%. In beef cows, factors such as inadequate nutrition and calf presence negatively impact the response of progesterone (P4)/estradiol-based ovulation synchronization protocols by interfering with GnRH release and consequently reducing LH pulsatility and final follicular development. In dairy cows, protocols based on GnRH and prostaglandin (Ovsynch) are the most widely used in the world. However, the efficiency of Ovsynch is dependent on the presence of a large follicle at the time of administration of the first GnRH. In these ovulation synchronization protocols, pre-synchronization protocols (Prostaglandins, Double Ovsynch and P4synch) are usually attempted in an effort to increase responses. Thus, the objective of this review was to discuss pre-ovulation synchronization strategies (administration of injectable P4 or energetic/protein supplementation or pre-synchronization with intra-vaginal progesterone devices) aiming to increase the LH pulsatility in beef cows or induce the formation of a GnRH-responsive follicle at the beginning of the Ovsynch protocol in dairy cows.

## Introduction

Currently, the TAI protocols in beef and dairy cattle are well established, in which pregnancy rates between 30 and 65% are observed (Baruselli *et al*., 2012; Sales *et al*., 2015; Wiltbank *et al*., 2015; Sales *et al*., 2016; Baruselli *et al*., 2017). However, the response to the TAI protocol (ovulation of largest follicle by inducer) based on estrogen and P4 is approximately 80% in *Bos indicus* lactating beef cows (Sales *et al*., 2012) and approximately 85% in dairy cows in GnRH-based protocols and PGF2α (Souza *et al*., 2008; Silva *et al*., 2018).

In *Bos indicus* lactating beef cows, a long period of postpartum anestrous is observed characterized by normal initial follicular growth sustained by the release of FSH, reduction of the final growth of the dominant follicle and, consequently, absence of ovulation (Baruselli *et al*., 2004). These changes in the final follicular growth are due to the reduction of LH pulsatility after follicle deviation due to the calf presence and the reduced availability of forage (Jolly *et al*., 1995; Yavas and Walton, 2000). In cows in which the nutritional requirement is not met because of low feed availability, deficient GnRH secretion and consequently, LH release are observed (Jolly *et al*., 1995; Montiel and Ahuja, 2005). The reduction of GnRH secretion occurs due to the negative feedback in the hypothalamus promoted by the increase in the concentrations of neuropeptide Y, NEFA and beta-hydroxybutyrate produced by the mobilization of body fat (Hess *et al*., 2005). In addition to the nutritional effects, the calf presence blocks the secretion of GnRH by the hypothalamus through the action of released endogenous opioids (Malven *et al*., 1986; Williams *et al*., 1996). Under this condition, part of the cows do not respond to the TAI protocol due to a drastic reduction in LH pulsatility observed mainly in primiparous cows (Sales *et al*., 2016) and in undernourished cows with low body condition score (Grimard *et al*., 1995; Diskin *et al*., 2003).In *Bos indicus* lactating cows it is necessary to stimulate the hypothalamus to produce GnRH to increase LH pulsatility which would allow for the final growth of the dominant follicle and ovulation. The positive effects of ovulation synchronization protocols in anestrous cows are mainly due to the stimulation of exogenous P4 on the pulsatility of GnRH and LH (Rhodes *et al*., 2002), allowing the ovulation of a pre- ovulatory follicle in the recent postpartum period (Baruselli *et al*., 2017). During the early postpartum period, progesterone reduces the expression of estradiol receptors in the hypothalamus by interfering with the hormone receptor-negative feedback in LH secretion (Ireland and Roche, 1982; Day, 2004). However, in underfed cows with body condition score <2.5 or primiparous, the final growth of the dominant follicle is lesser, resulting in small follicles at the time of TAI (Sales *et al*., 2016). Thus, in females that do not respond to the TAI protocol, the period of exposure to P4 during the ovulation synchronization protocol may not be sufficient to increase the LH pulsatility needed for ovulation to occur. Therefore, supplementation with P4 prior to the protocol is an alternative to improve thereproductive efficiency of beef cows submitted to TAI protocols (Simões *et al*., 2018). In addition to the effects of P4 in *Bos indicus* beef cows, energy/protein supplementation may increase the reproductive efficiency of beef cows submitted to the TAI protocol (Peres *et al*., 2016; Orlandi *et al*., 2018).

In dairy cows, the protocols based on GnRH and PGF2α are predominant due to the ban on the use of esters of estradiol in some countries. The standard protocol used based on GnRH and PGF2α is Ovsynch (Pursley *et al*., 1995). In spite of attending to the ovulation synchronization assumptions, there is low synchronization efficiency (64%) in this protocol when administered on a random day of the estrous cycle (Vasconcelos *et al*., 1999). The best results to start the Ovsynch protocol is between the 5th and 12th day of the estrous cycle, as at this period is common to have a dominant follicle responsive to the GnRH treatment (Vasconcelos *et al*., 1999). Thus, pre-synchronization protocols are used to increase the proportion of cows with a responsive dominant follicle at the first GnRH of the ovsynch protocol (Moreira *et al*., 2001). Among the pre-synchronization protocols, Double-Ovsynch has presented a better synchronization result, with ovulation rates at the first GnRH of around 82% and pregnancy rates of 49.7% (Souza *et al*., 2008). However, some limitations (long protocol of 28 days and many animal handling) prevent extensive use of this protocol. In addition, to stimulating LH pulsatility, P4 (single intravaginal P4 devices) may be an alternative to induce the formation of large follicles that responds to the first GnRH of the Ovsynch protocol (Silva *et al*., 2018). Cows with P4 devices develop follicular persistence due to absence of pre-ovulatory peak of LH and maintenance of sub luteal concentrations of progesterone (Cerri *et al*., 2009). Persistent follicles are capable of ovulating after long periods (15 days) of progestogen blocking (Chebel *et al*., 2006). Thus, the persistent follicle can be used as a pre-synchronization method for the Ovsynch protocol due to the constant follicular development and ovulatory capacity. Thus, the objective of this review was to propose strategies that increase the response to ovulation synchronization protocols in beef and dairy cows using P4 or protein/energy supplementation pre-protocol of TAI, aiming to increase LH pulsatility or induce a GnRH-responsive follicle at the beginning of the ovulation synchronization protocol.

## Strategies to increase LH pulsatility prior to TAI protocols

Postpartum anestrous in cows is caused in part by a reduction in LH pulsatility after follicular divergence (Yavas and Walton, 2000). This gonadotropin depletion is caused by the strong negative feedbacks from progesterone and estrogens in late pregnancy. The period of anovulatory anestrus varies between cows and milk production level. In dairy cows, the interval between calving and first ovulation ranges from 19 to 22 days (Darwash *et al*., 2010). However, in dairy cows on grazing systems, this interval may increase up to 43 days (McDougall *et al*., 1995). In *Bos indicus* beef cows raised in a continuous grazing system, longer postpartum anestrous periods are observed (>100 days ; Baruselli *et al*., 2004). Under this management system, between 5 and 15% of the cows are cycling at the beginning of the breeding season (Sales *et al*., 2011; Baruselli *et al*., 2017). In this regards, strategies for stimulation of GnRH-induced LH secretion during early postpartum to reduce anestrous period were attempted, such as P4 (Simões *et al*., 2018) and energetic/protein (Peres *et al*., 2016; Orlandi *et al*., 2018) supplementation.

## Progesterone

Progesterone increases LH pulsatility by reducing the expression of estrogen receptors in the hypothalamus, decreasing negative feedback for GnRH production and release (Anderson and Day, 1998; Day, 2004). Thus, treatment with P4 in anestrous cows increased follicular fluid estradiol concentration due to increased LH pulsatility and its LH receptors on granulosa and theca cells in pre-ovulatory follicles (Inskeep *et al*., 1988; Rhodes *et al*., 2002). Some studies have shown that the use of P4 stimulates cyclicity in lactating dairy cows (Fike *et al*., 1997; Lucy *et al*., 2001). Recently, our research group conducted studies to evaluate the effect of injectable P4 (P4i) on the reproductive efficiency of lactating *Bos indicus* cows submitted to TAI. In the first study (Simões *et al*., 2018) the effect of previous exposure to injectable progesterone (P4i) in TAI protocols on follicular growth and pregnancy rate of *Bos indicus* lactating cows was evaluated. In this study, 420 lactating anestrous Nelore cows were used. Cows were divided into one of three experimental groups (Control, P4, and P4GnRH), 10 days before (D-10) the beginning of the P4 and estrogen-based ovulation synchronization protocol (Sales *et al*., 2015). In the control group, cows were only submitted to the protocol based on P4 and estrogen. In the P4i group, cows received 150 mg of P4i (Sincrogest Injectable®, Ouro Fino, Brazil) intramuscularly on D-10 and were submitted to the same ovulation synchronization protocol as in the Control group. In the P4iGnRH group, cows received the same treatments of the P4 group associated with the administration of 10μg of buserelin (Sincroforte®, Ouro Fino, Brazil) on D0. In this study, the P4i treatment increased the follicular diameter at the beginning of the TAI protocol and on the day of removal of the P4 device. In addition, cows receiving pre-protocol P4 were 1.68 times more likely to become pregnant after TAI than the control group (Tab. 1). In *Bos taurus* beef cows (Simões, unpublished data), receiving P4i treatment previous to TAI protocol increased P/AI [Control 45.6% (118/259) and P4i 54.8% (142/259); P = 0.03]. In another study (Santos *et al*., 2018) using 988 lactating Nelore cows in adequate body condition score (~3.0), a P4 treatment preceding the ovulation synchronization protocol did not improve pregnancy rate [Control 64.7% (322/498) and P4i 62.9% (308/490); P = 0.55] and cyclicity 30 days after TAI [Control 39.8% (70/176) and P4i 39.6% (72/182) P = 0.78]. Thus, probably in cowswith adequate body condition LH pulsatility in postpartum should allow growth and ovulation of a preovulatory follicle. This difference in fertility after P4 treatment is probably due to the body condition of the animals in the different studies. In the study by Simões *et al*. (2018), the cows were nutritionally impaired which resulted in low body condition scores. Nutritionally deficient cows have lower postpartum LH pulsatility due to the formation of metabolites (NEFA, Beta-hydroxybutyrate and acetate), endorphins and peptides (mainly neuropeptide Y) known to produce negative feedback blocking hypothalamic GnRH (Hess, 2005). Thus, treatment with P4 prior to ovulation synchronization protocols may have increased LH secretion (Anderson and Day, 1998; Day, 2004), which resulted in higher pregnancy rates.

**Table 1:** Effects of exposure to injectable progesterone previous to TAI protocol on follicular growth, CL diameter and ovulation rate of suckled Nelore cows.

	Control	P4i	P4iGnRH	P
Diameter (mm)				
LF on Day 0 (mm)	10.9 ± 0.2^b^	12.7 ± 0.3^a^	12.6 ± 0.4^a^	0.001
LF on Day 8 (mm)	9.7 ± 0.2^b^	10.4 ± 0.2^a^	9.9 ± 0.2^ab^	0.05
LF on Day 10 (mm)	12.6 ± 0.3	13.0 ± 0.3	12.6 ± 0.3	0.21
CL on Day 24 (mm)	19.7 ± 0.4^ab^	20.1 ± 0.4^a^	18.5 ± 0.4^b^	0.001
Follicular growth rate (mm/day)	1.4 ± 0.1	1.4 ± 0.1	1.3 ± 0.1	0.34
Ovulation rate (%)	78.2 (104/133)	80.3 (110/137)	75.2 (106/141)	0.62
CL presence on Day 8 (%)	0.0 (0/136)^b^	0.0 (0/140)^b^	26.4 (38/144)^a^	0.001
P/AI	34.9 (78/223)^b^	45.9 (105/229)^a^	40.6 (93/229)^ab^	0.01

Abbreviations: LF - largest follicle; CL - Corpus Luteum; P/AI - pregnancy per timed-AI. Control group - cows were only submitted to the conventional protocol based on P4 and estrogen P4i group - cows received 150mg of progesterone injectable intramuscularly 10 days before initiation of the ovulation synchronization protocol (D-10). P4iGnRH group - cows received the same treatments of the P4 group associated with the administration of 10μg of buserelin on D0. Different letters (a≠b) in the same line differ (P < 0.05; Simões *et al*., 2018).

Based on the benefits reported here, we hypothesize that the prior use of P4 could replace eCG in TAI protocols (Simões, unpublished data). Research emphasizes the importance of treatment with eCG to increase both ovulation and pregnancy rates in TAI protocols (Baruselli *et al*., 2004; Sales *et al*., 2011; Sales *et al*., 2016). As shown previously, eCG has positive effects on recently calved anestrous cows (postpartum period less than 2 months) in animals with compromised body condition (Sales *et al*., 2011) and in cows with dominant follicle growth impairment due to high levels of progesterone at the end of the ovulation synchronization treatment (Baruselli *et al*., 2004). Despite the great benefits of eCG, the use of this gonadotrophin is banned in some countries and a resistance front has emerged because of the way it is extracted from mares. In addition, eCG has no activity pattern and its cost is extremely high. In the Simões study (unpublished data), 600 lactating Nelore multiparous cows were used and distributed in four experimental groups. In the control group (n = 150), cows were submitted to an ovulation synchronization protocol based on P4 and estrogen (D0 - 2mg estradiol benzoate (EB) + P4 device; D8 - withdrawal P4 device + 1mg estradiol cypionate (EC) + 500ug Cloprostenol; D10 - TAI). In the eCG group, cows were submitted to the same ovulation synchronization protocol of the Control group associated with the administration of 300 IU of eCG in D8. In the P4i group, the cows were submitted to the same TAI protocol of the control group associated with the administration of 150mg of injectable P4 (Sincrogest injectable®) 10 days before the initiation of the ovulation synchronization protocol. In the P4ieCG group, cows underwent the same TAI protocol from the Control group associated with the administration of 150mg of injectable P4 and 300UI of eCG in D8. The association of eCG with P4i prior to the protocol increased follicular diameter at day 10 of the TAI protocol. However, the use of P4i without the administration of eCG resulted in a lower pregnancy rate. However, there was an additive gain in pregnancy rate with the association of eCG and P4i prior to the protocol, similar to that previously observed in *Bos indicus* cows (Simões *et al*., 2018). Thus, in *Bos indicus* cows, P4 treatment prior to the TAI protocol is not a viable alternative to replace eCG.

## Energetic and protein supplementation

**Figure 1:**
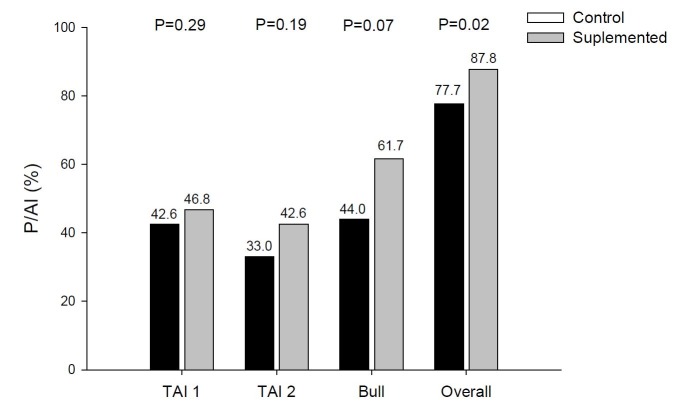
P/AI during a 110-day breeding season of lactating Nelore cows supplemented with energy and protein diet at the end of the dry period (Orlandi *et al*., 2018).

Under feed restriction cows mobilize body energy reserves, resulting in increases in the concentration of neuropeptide Y (McShane *et al*., 1993) and NEFA from mobilization of body energy reserves (DiCostanzo *et al*., 1999) which, in turn, block the secretion of GnRH and, consequently, the release of LH (Schillo, 1992). In addition, cows in negative energetic balance have high concentrations of β-hydroxybutyrate and low glucose concentrations that reduce GnRH secretion by the hypothalamus (Mulliniks *et al*., 2013). Therefore, adequate nutrition during the pre-partum period and the amount of dry matter available in postpartum are key elements for the return to cyclicity in dairy and beef cows (Crowe *et al*., 2014). Studies have shown that cows with adequate body condition pre and postpartum have greater fertility after calving (Sa Filho *et al*., 2009; Ayres *et al*., 2014) and that energy and/or protein supplementation increases the conceptionrate (Pescara *et al*., 2010). In a recent study by our research group (Orlandi *et al*., 2018), the effect of energy and protein supplementation on follicular growth and pregnancy rate of *Bos indicus* lactating cows was evaluated. In this study, 342 *Bos indicus* (Nelore) cows in anestrus were distributed in Control (non-supplemented cows) and Supplement (cows received 2.5 kg/day of an energy/protein supplement with 26.5% CP and 76.5% NDT for 26 days) groups. Supplementation was initiated 12 days prior to a standard P4 and estradiol based-TAI protocol and maintained for 14 days. After the first TAI, the non-pregnant cows were resynchronized and 10 days after the second TAI were exposed to Nelore clean-up bulls until the end of the breeding season, which lasted for 110 days. The diameter of the largest follicle at D0, D8, D10, CL diameter at D14 and ovulation rate were higher (P < 0.05) in the Supplement group. In addition, there were no differences (P > 0.05) between the treatments for P/AI at 1^st^ and 2^nd^ TAI or after the clean-up bull. However, the pregnancy rate at the end of the breeding season was greater in the Supplement group (P = 0.02; Fig. 1).

In another supplementation study in beef cows (Peres *et al*., 2016), the effect of corn-based supplementation was evaluated for 41 days. The supplementation started on the first day of insertion of the P4 device (D0) in the TAI protocol and remained until pregnancy check (D41). In this study two experiments were carried out to evaluate the hormonal profile and the fertility of Nelore females. In the study, the three-way TAI protocol with 11-day duration was used. In experiment 1,681 primiparous cows averaging 2.84 in BCS were used to evaluate the concentrations of IGF-1, leptin and GH and in experiment 2, 2395 Nelore females (648 heifers, 635 primiparous and 1112 multiparous) were submitted to the TAI protocol to evaluate fertility. In both experiments cows were distributed into two groups, Control (not supplemented) and Supplemented (1.0kg/cow/day of corn from D0 to D11 and 2.2kg/cow/day from D11 to D41). Both groups grazed on pastures with ad libitum access to water and mineral and TAI protocols started 35 days post calving. In experiment 1, the higher concentration of IGF-1 at TAI (138.4 *vs* 130.8ng/mL) and lower concentration on the day of the pregnancy diagnosis (135.5 *vs* 141.5ng/mL) were observed in the supplemented cows. In this study, cows with higher concentrations of IGF-1 at the time of TAI and leptin / GH at the beginning of the protocol had higher pregnancy rates, demonstrating that supplementation during the protocol may increase the pregnancy rate due to an increase in the concentration of IGF-1. In experiment 2, although corn-based supplementation did not interfere with the results of the first TAI (Control - 50.9% and Supplemented - 52.4%), there was a trend of higher pregnancy rate in the second TAI in supplemented cows (44.3% *vs*. 38.5%). In addition, primiparous cows had higher pregnancy rate at the end of the breeding season (77.8% vs. 65.7%), showing cumulative/late effects similar to those observed in Orlandi *et al*. (2018). However, in multiparous, the final pregnancy rate was lower in the supplemented group (87.0% vs. 92.0%). These results make it difficult to understand the effects of energy supplementation on beef cows during and after the TAI protocol. Thus, the positive effects of nutrition may occur due to changes in hormone concentrations (insulin and IGF-I) and metabolites (glucose, cholesterol and beta-hydroxybutyrate) related to reproductive efficiency(Beam and Butler, 1998; Ospina *et al*., 2010; Mulliniks *et al*., 2013; Samadi *et al*., 2013). Thus, short duration(<30 days) energy and protein supplementation 12 days before TAI sufficient to animal maintenance increased fertility lactating beef cows in the post postpartum period, being an interesting alternative.

## Strategies to increase response to the first GnRH of the Ovsynch protocol

Ovsynch is the standard GnRH/PGF2α-based protocol (Pursley *et al*., 1995) where a first GnRH is given on a random day of the estrous cycle (D0). Seven days later, PGF2α (D7) is given and 48 hours later, a second GnRH. Cows are inseminated 16 hours after the second GnRH. Although it meets the three premises of ovulation synchronization, this protocol presents low efficiency (64%) if administered in animals on a random day of the estrous cycle (Vasconcelos *et al*., 1999). In this study, ovulation rate was higher in cows that received the first GnRH of the Ovsynch protocol between days 5 - 9 and 17 - 21 days of the estrous cycle. In addition, there was a higher ovulation rate to the second GnRH of the Ovsynch protocol when the animals responded to the first GnRH (Vasconcelos *et al*., 1999). However, cows that did not respond to the first GnRH had a longer period of dominance of the ovulatory follicle (follicular persistence), compromising oocyte quality and early embryo development (Cerri *et al*., 2009). Such changes in follicular dynamics resulted in a lower pregnancy rate (Chebel *et al*., 2006). Thus, pre-synchronization protocols have been used to increase the response to the first GnRH of the Ovsynch protocol, (Moreira *et al*., 2001; Souza *et al*., 2008).

The first pre-synchronization protocol used two PGF2α with a 14-day interval, followed by Ovsynch 12 days after the second PGF2α (termed Presynch-Ovsynch; Moreira *et al*., 2001). Pre-synchronization in this study increased the conception rate (37% *vs* 49%) in heifers by 12 percentage points and other authors observed an increase of 18 percentage points in cyclic lactating cows (25% *vs* 43%; El-Zarkouny *et al*., 2001). In another study, using a similar protocol (twelve day intervals between PGF2α injections) conception rates at 42 days of gestation was 49.6% for the cows in the Presynch group and 37.3% for cows in the Ovsynch group (Navanukraw *et al*., 2004). Thus, such favorable results are attributed to a larger number of animals in the optimal phase of the estrous cycle receiving the Ovsynch protocol. However, only cyclic cows can benefit from the program with two PGF2α since the response depends on the presence of responsive corpus luteum (Chebel *et al*., 2006). Another limitation of the effectiveness of the Presynch-Ovsynch protocol would be the lack of precision in follicular synchronization and luteal stages, due to estrous variability and ovulation after PGF2α treatments (Ayres *et al*., 2013).

Among the pre-synchronization protocols, Double Ovsynch (Ovsynch protocol is performed as a pre-synchronization tool) has achieved better synchronization results (Souza *et al*., 2008). Double-Ovsynch comprises two Ovsynch protocols seven-days apart, with TAI after the last GnRH of the second protocol. Double-Ovsynch increases the ovarian response to hormone treatment and P4 concentrations during the Ovsynch of the TAI (Souza *et al*., 2008). In this study, 28% more cows with high progesterone (>3ng/mL) were observed at the time of PGF2α in the Double-Ovsynch group (78.1% *vs* 52.3%) when compared to the group treated with two PGF2α. In addition, there was a high ovulation rate at the first GnRH (82%) and a satisfactory pregnancy rate (49.7%). In another study (Herlihy *et al*., 2012), Double-Ovsynch reduced the proportions of primiparous and multiparous cows with low circulating P4 concentrations compared to Presynch-Ovsynch treated cows (3.3 *vs* 19.7% in primiparous and 8.8 *vs* 31.9% in multiparous). Cows with low concentrations of P4 at the time of PGF2α administration are more likely to have premature luteolysis, with consequent peak LH and ovulation prior to administration of the second Ovsynch GnRH (Vasconcelos *et al*., 1999). In both studies, the Double-Ovsynch protocol increased Ovsynch fertility compared to Presynch-Ovsynch. The ovulatory response to the first Ovsynch GnRH increases the circulating concentrations of progesterone and allows the development of the dominant follicle less variable and closer to the ideal size at the time of the second GnRH (Bello *et al*., 2006; Giordano *et al*., 2012). Increased circulating concentrations of P4 during follicular development may decrease LH pulsatility, possibly increase dominant follicle competence, oocyte and uterine environment qualities (Mihm *et al*., 1994; Revah and Butler, 1996). Other studies also related ovulation to the first Ovsynch GnRH and the presence of CL at the time of PGF2α with higher pregnancy rates at 30 and 60 days post artificial insemination (Vasconcelos *et al*., 1999; Chebel *et al*., 2006). However, such a protocol is too long (28 days) and difficult to implement on farm. Thus, there is still the need for the development of more practical and shorter pre-synchronization protocols.

**Figure 2:**
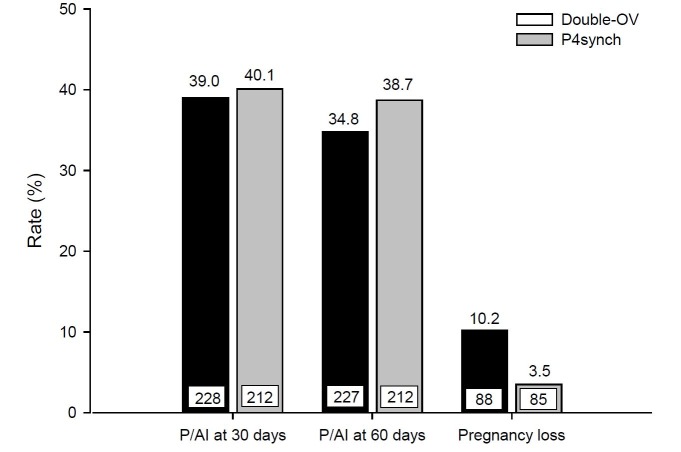
Effect of the presynchronization protocol (Double-Ov and P4synch) on the P/AI at 30 and 60 days and pregnancy loss (P > 0.05) in lactating crossbred dairy cows (Silva *et al*., 2018).

Recently our research group developed a pre-synchronization protocol using a P4 sustained-release vaginal device (Silva *et al*., 2018) to induce a persistent dominant follicle to increase the response to the first GnRH of the Ovsynch protocol. In the experiment, 440 dairy cows (345 Holstein-Zebu crossbreds and 95 Holsteins) were randomly assigned to Double Ovsynch (Double-Ov; Souza *et al*., 2008) and P4synch. The P4synch protocol consisted of insertion of an intravaginal P4 device 10 days prior to the initiation of the Ovsynch protocol (D-10) and withdrawing the device on the day of PGF2α administration of the Ovsynch (D7) protocol. All cows were inseminated 16 hours after the second dose of GnRH from the Ovsynch protocol. No differences were observed between the groups for the pre-synchronization rate variables [presence of follicles with more than 12mm in the D0; P = 0.66), follicular diameter at the 1^st^ GnRH (P = 0.28), ovulation rate at 1^st^ GnRH (P = 0.50), synchronization rate (P = 0.84), follicular diameter at the 2^nd^ GnRH (P = 0.48), ovulation rate in the 2^nd^ GnRH (P = 0.48) and the diameter of the CL in D24 (P = 0.19)]. However, thepresence of CL on D0 was higher (P = 0.03) in the Double Ovsynch group (Tab. 2). In addition, there was no difference in pregnancy rates at 30 (P = 0.85), at 60 days of gestation (P = 0.41) and in gestational losses at 30 and 60 days of gestation (P = 0.13; Fig. 2). There was no difference in the percentage of cows with P4 <1ng/mL at D0 [Double-Ov 13.6% (3/22) and P4synch 5.0% (1/20); P = 0.37], for percentage of cows with P4 >1ng/mL in D7 (Double-Ov 77.3% and P4synch 95.0%; P = 0.14) and for P4 concentration in D24 (Double-Ov 4.7 ± 0.6 and P4synch 5.9 ± 0.9 ng/mL, P = 0.84). The P4synch protocol has the same reproductive efficiency as the Double Ovsynch protocol in lactating dairy cows. In another study by our research group (Lima, unpublished data), we compared the P4synch protocol with the protocol based on estrogen and P4 (Ferreira *et al*., 2013). In this study, similar results were reported between the P4synch and the P4-estradiol-based protocols (Tab. 3). Thus, P4synch may be an efficient alternative for ovulation synchronization in dairy cows.

**Table 2:** Effect of presynchronization (Double Ovsynch and P4synch) on the follicular dynamics of lactating crossbred dairy cows submitted to the Ovsynch protocol.

	Double-Ov	P4synch	P
Rates (%)			
Presynchronization	94.2 (49/52)	92.0 (46/50)	0.66
CL on Day 0	57.7 (30/52)	36.0 (18/50)	0.03
Ovulation to 1^st^ GnRH	86.3 (44/51)	81.2 (39/48)	0.50
Follicular persistence	5.9 (03/52)	14.3 (07/49)	0.20
Synchronization of Day 9	84.6 (44/52)	86.0 (43/50)	0.84
Ovulation to 2^nd^ GnRH	90.9 (40/44)	86.0 (37/43)	0.48
Diameters (mm)			
LF on Day 0	17.2 ± 07	18.6 ± 0.8	0.28
LF on Day 9	17.6 ± 0.5	17.9 ± 0.4	0.48
CL on Day 24	27.9 ± 0.7	29.4 ± 0.8	0.19

Abbreviations: LF, largest follicle; CL, Corpus Luteum. a) Presynchronization: presence of follicle >12mm on D0. b) Follicular persistence: presence of follicle >12mm on D0, absence of CL on D7 and follicle >20mm on D9. c) Synchronization: presence of a follicle >12 mm. The P4synch protocol consisted of insertion of an intravaginal P4 device 10 days prior to the initiation of the Ovsynch protocol (D-10) and withdrawing the device on the day of PGF2α administration of the Ovsynch (D7) protocol (Silva *et al*., 2018).

**Table 3:** Effect of protocols (P4E2 and P4synch) on the follicular dynamics and fertility of lactating dairy cows.

	P4E2	P4synch	P
Rates (%)			
Presynchronization	73.9 (34/46)	97.8 (45/46)	0.01
CL on Day 0	80.4 (37/46)	37.0 (17/46)	0.001
Ovulation to 1^st^ GnRH	65.2 (30/46)	65.2 (30/46)	0.99
Follicular persistence	8.7 (4/46)	15.2 (7/46)	0.34
Synchronization on induction	76.1 (35/46)	80.4 (37/46)	0.61
Diameters (mm)			
LF on Day 0	15.0 ± 0.8	21.0 ± 0.8	0.001
LF on induction	13.9 ± 0.7	17.6 ± 0.6	0.001
LF on TAI	15.2 ± 0.7	17.2 ± 0.8	0.05
P/AI	37.4 (67/179)	42.4 (72/170)	0.35

Abbreviations: LF, largest follicle; CL, Corpus Luteum. a) Presynchronization: presence of follicle >12mm on D0. b) Follicular persistence: presence of follicle >12mm on D0, absence of CL on D7 and follicle >20mm on D9. c) Synchronization on induction: presence of a follicle >12 mm. The P4synch protocol consisted of insertion of an intravaginal P4 device 10 days prior to the initiation of the Ovsynch protocol (D-10) and withdrawing the device on the day of PGF2α administration of the Ovsynch (D7) protocol (Lima *et al*., unpublished).

## Conclusion

The TAI protocols in beef and dairy cows are well established, but the response to protocols ranges from 70 to 90%. Currently, there are strategies to increase protocol response in cows with LH release impairment. The P4i strategy brought significant increase in fertility in Bos indicus and Bos taurus beef cows. Another strategy that has improved TAI results in Bos indicus beef cows is energy/protein supplementation before and during the protocol. In addition, the use of intravaginal P4 device is an efficient alternative of pre-synchronization to the Ovsynch protocol in dairy cows.

Despite these results, more studies are necessary to confirm these findings, especially in energy/protein supplementation.
